# Updated checklist of the extant Chondrichthyes within the Exclusive Economic Zone of Mexico

**DOI:** 10.3897/zookeys.774.25028

**Published:** 2018-07-12

**Authors:** Nicolás Roberto Ehemann, Lorem del Valle González-González, Jorge Guillermo Chollet-Villalpando, José De La Cruz-Agüero

**Affiliations:** 1 Instituto Politécnico Nacional – Centro Interdisciplinario de Ciencias Marinas (CICIMAR–IPN), Colección Ictiológica, Avenida IPN s/n, Colonia Playa Palo Santa Rita, La Paz, Baja California Sur, 23096, México; 2 Actual address: Instituto de Ecología, A.C. – INECOL, Biodiversidad y Sistemática, Carretera Antigua a Coatepec 351, Colonia El Haya, Xalapa, 91070, Veracruz, México

**Keywords:** chimaeras, elasmobranchs, rays, sharks, systematics, taxonomy

## Abstract

The checklist presented in this study includes the latest taxonomic and systematic modifications and updates (early 2018) for the Chondrichthyes that inhabit the Exclusive Economic Zone (EEZ) of Mexico. The list is based on a literature review of field-specific books, scientific publications and database information from collections and museums worldwide available online such as, the Ocean Biogeographic Information System (OBIS), Global Biodiversity Information Facility (GBIF), Encyclopedia of Life (EOL), iSpecies, FishBase and the National Biodiversity Information System (SNIB–CONABIO). Information was cross-referenced with digital taxonomic systems such as the Catalog of Fishes of the California Academy of Sciences, the World Register of Marine Species (WoRMS), and the Integrated Taxonomic Information System (ITIS). There is a total of two subclasses two divisions, 13 orders, 44 families, 84 genera, and 217 species that represent approximately 18% of all living and described species of chondrichthyans worldwide. For the Mexican Pacific and the Gulf of California, 92 species of chondrichthyans are listed compared to 94 species for the Gulf of Mexico and the Caribbean Sea. Additionally, 31 species listed occur on both coasts of Mexico. The species richness of the Mexican chondrichthyans will surely continue to increase, due to the exploration of deep-water fishing areas in the EEZ.

## Introduction

The natural history of the Chondrichthyes (chimaeras, sharks, skates, and rays) inhabiting the waters of the maritime territory and the adjacent and oceanic zones of Mexico, (referred hereinafter as the Exclusive Economic Zone (EEZ)) has always been a difficult issue to address. The causes of the underestimation or overestimation of these species can be diverse (Del Moral-Flores et al. 2015a) and are typically attributed to i) incorrect taxonomic identifications, ii) undetected synonyms, iii) inaccurate or incorrect curatorial records (e.g., date of collection, location, coordinates), iv) use of obsolete information and, v) absence of updated taxonomic lists.

Recent chondrichthyans studies have led to various taxonomic and systematic readjustments, name substitutions, new gender-specific combinations and description of new species. New taxonomic arrangements are based on combined conventional morphology, geometric morphometrics and DNA studies (e.g., Del Moral-Flores et al. 2015b, Acero et al. 2016, Last et al. 2016a, Last et al. 2016b, Last et al. 2016c, Last et al. 2016d, White and Naylor 2016).

The current systematic checklist of chimaeras, sharks, rays, and skates from the Mexican EEZ, incorporates the newest taxonomic and systematic proposals for chimaeras and sharks (Ebert et al. 2013, Weigmann 2016, 2017), skates and rays (Last et al. 2016a, b, c, d, Weigmann 2016, 2017) and follows, with some nomenclatural modifications, the Chondrichthyes classifications made by Weigmann 2016. The present inventory also includes information provided since last century until last year regarding the chondrichthyans of Mexico and Mexican endemic species (e.g., Castro-Aguirre 1965, Castro-Aguirre et al. 1996, Espinosa-Pérez et al. 2004, Palacios-Salgado et al. 2012, Del Moral-Flores and Pérez-Ponce de León 2013, Del Moral-Flores et al. 2015a, Del Moral-Flores et al. 2016).

In Mexico, an updated inventory of the natural resources relevant to Chondrichthyes is necessary, and many ecological relationships are also unknown. This information is imperative for developing environmental or biogeographic theory and for explaining scientific implications of the availability of resources (Siqueiros-Beltrones and De La Cruz-Agüero 2004). Systematic lists are thus a fundamental requirement for the decision-making process in the evaluation of the changes in biodiversity derived from anthropogenic factors and consequently, for the establishment of regional systems and priority areas with regard to the conservation of Mexican chondrichthyans. Due to the constant updating of taxonomic and systematic information for Chondrichthyes, the primary objective of this study is to provide an updated list (early 2018) of the chimaeras, sharks, skates, and rays, living in the EEZ of Mexico.

## Materials and methods

**Figure 1.**
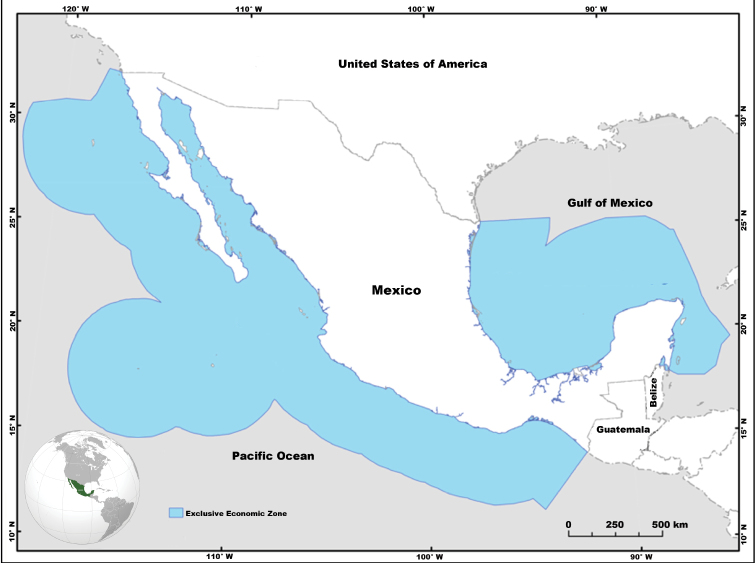
Map showing the Exclusive Economic Zone (EEZ) of Mexico. The zone comprises, including islands and territorial sea, approximately 3,150,000 km^2^. Modified from CONABIO (2011), available at: http://www.conabio.gob.mx/informacion/gis/layouts/contdv250_zeemgw.png

First, the Weigmann taxonomic checklists (2016, 2017) were consulted to select the species that were registered in the areas defined by the latter author (see also IHO 1953) as northeastern Pacific (NEP, Canada to Panama) and northwestern Atlantic (NWA, eastern United States of America to the southern Caribbean Sea). In this geographical framework, all chondrichthyan species with a distribution identified within the Mexican EEZ were included. The selected taxocenoses were contrasted and compared with the biological stocks recorded in the Ichthyological Collection (IC) (http://coleccion.cicimar.ipn.mx/) of the Centro Interdisciplinario de Ciencias Marinas (marked with an asterisk in Table 1). The Mexican EEZ is the area of ocean extending 200 nautical miles (370.4 km) from the coast (DOF 1976). This geographic boundary refers to a political delineation rather than an ecological boundary (Figure 1).

**Table 1.** A systematic listing of the chondrichthyans inhabiting the Exclusive Economic Zone of Mexico (EEZ). The checklist was depurated from references, field books, catalogued specimens and online databases (see text) about the distribution of Chondrichthyes in the littoral areas of the Pacific Ocean (including the Gulf of California) and the Gulf of Mexico (including the Caribbean Sea). The systematic arrangement is based on Weigmann (2016), with some modifications for the batomorphs cited by Last et al. (2016a). The species are listed in alphabetical order. (*) = Species with specimens cataloged in the Ichthyological Collection (IC) of CICIMAR–IPN (http://coleccion.cicimar.ipn.mx/). (†) = New valid taxa according to Weigmann (2017) and Last et al. (2016a), taking into account that when a genus is designated, all species are included. (?) = Species whose validity requires further investigation. (West C.) = west coast of the EEZ of Mexico. (East C.) = east coast of the EEZ of Mexico.

Subclass	Order	Family	Genus	Species	West C.	East C.
**Holocephali**	**Chimaeriformes**	Rhinochimaeridae	*Harriotta*	*Harriotta haeckeli* Karrer, 1972	√	
				*Harriotta raleighana* Goode & Bean, 1895	√	
			*Rhinochimaera*	*Rhinochimaera atlantica* Holte & Byrne, 1909		√
		Chimaeridae	*Hydrolagus*	*Hydrolagus alberti* Bigelow & Schroeder, 1951		√
				*Hydrolagus colliei* * (Lay & Bennett, 1839)	√	
				*Hydrolagus macrophthalmus* De Buen, 1959	√	
				*Hydrolagus melanophasma* * James, Ebert, Long & Didier, 2009	√	
				*Hydrolagus mirabili*s (Collett, 1904)		√
**Euselachii**						
**Infraclass**						
**Elasmobranchii**						
**Division**	**Order**	**Family**	**Genus**	**Species**	**West C.**	**East C.**
**Selachii**	**Heterodontiformes**	Heterodontidae	*Heterodontus*	*Heterodontus francisci* * (Girard, 1855)	√	
				*Heterodontus mexicanus* * Taylor & Castro-Aguirre	√	
	**Orectolobiformes**	Ginglymostomatidae	*Ginglymostoma*	*Ginglymostoma cirratum* (Bonnnaterre, 1788)		√
				*Ginglymostoma unami* * Del Moral-Flores, Ramírez-Antonio, Angulo & Pérez-Ponce de León, 2015	√	
		Rhincodontidae	*Rhincodon*	*Rhincodon typus* Smith, 1828	√	√
	**Lamniformes**	Alopiidae	*Alopias*	*Alopias pelagicus* Nakamura, 1935	√	
				*Alopias superciliosus* Lowe, 1841	√	√
				*Alopias vulpinus* (Bonnaterre, 1788)	√	√
**Selachii**	**Lamniformes**	Cetorhinidae	*Cetorhinus*	*Cetorhinus maximus* (Gunnerus, 1765)	√	√
		Lamnidae	*Carcharodon*	*Carcharodon carcharias* (Linneaeus, 1758)	√	√
			*Isurus*	*Isurus oxyrinchus* Rafinesque, 1810	√	√
				*Isurus paucus* Guitart, 1966	√	√
			*Lamna*	*Lamna ditropis* Hubbs & Follett, 1947	√	
		Megachasmidae	*Megachasma*	*Megachasma pelagios* Taylor, Compagno & Struhsaker, 1983	√	√
		Odontaspididae	*Carcharias*	*Carcharias taurus* Rafinesque, 1810		√
			*Odontaspis*	*Odontaspis ferox* (Risso, 1810)	√	√
				*Odontaspis noronhai* (Maul, 1955)	√	
		Pseudocarchariidae	*Pseudocarcharias*	*Pseudocarcharias kamoharai* * (Matsubara, 1936)	√	√
	**Carcharhiniformes**	Carcharhinidae	*Carcharhinus*	*Carcharhinus acronotus* (Poey, 1860)		√
				*Carcharhinus albimarginatus* (Rüppell, 1837)	√	
				*Carcharhinus altimus* (Springer, 1950)	√	√
				*Carcharhinus brachyurus* (Günther, 1870)	√	
				*Carcharhinus brevipinna* (Müller & Henle, 1839)		√
				*Carcharhinus cerdale* Gilbert, 1898	√	
				*Carcharhinus falciformis* (Müller & Henle, 1839)	√	√
				*Carcharhinus galapagensis* (Snodgrass & Heller, 1905)	√	
				*Carcharhinus isodon* (Müller & Henle, 1839)		√
				*Carcharhinus leucas* (Müller & Henle, 1839)	√	√
				*Carcharhinus limbatus* * (Müller & Henle, 1839)	√	√
				*Carcharhinus longimanus* (Poey, 1861)	√	√
				*Carcharhinus melanopterus* (Quoy & Gaimard, 1824)		√
				*Carcharhinus obscurus* (Lesueur, 1818)	√	√
				*Carcharhinus perezii* (Poey, 1876)		√
				*Carcharhinus plumbeus* (Nardo, 1827)		√
				*Carcharhinus porosus* * (Ranzani, 1839)	√	√
				*Carcharhinus signatus* (Poey, 1868)		√
			*Galeocerdo*	*Galeocerdo cuvier* (Péron & Lesueur, 1822)	√	√
			*Nasolamia*	*Nasolamia velox* * (Gilbert, 1898)	√	
**Selachii**	**Carcharhiniformes**	Carcharhinidae	*Negaprion*	*Negaprion brevirostris* (Poey, 1868)	√	√
			*Prionace*	*Prionace glauca* * (Linnaeus, 1758)	√	√
			*Rhizoprionodon*	*Rhizoprionodon longurio* * (Jordan & Gilbert, 1882)	√	
				*Rhizoprionodon porosus* (Poey, 1861)		√
				*Rhizoprionodon terraenovae* (Richardson, 1836)		√
			*Triaenodon*	*Triaenodon obesus* (Rüppell, 1837)	√	
		Pentanchidae	*Apristurus*	*Apristurus brunneus* (Gilbert, 1892)	√	
				*Apristurus kampae* Taylor, 1972	√	
				*Apristurus laurussonii* (Saemundsson, 1922)		√
				*Apristurus nasutus* de Buen, 1959	√	
				*Apristurus parvipinnis* Springer & Heemstra, 1979		√
				*Apristurus riveri* Bigelow & Schroeder, 1944		√
			*Cephalurus*	*Cephalurus cephalus* (Gilbert, 1892)	√	
			*Galeus*	*Galeus arae* (Nichols, 1927)		√
				*Galeus piperatus* Springer & Wagner, 1966	√	
			*Parmaturus*	*Parmaturus campechiensis* Springer, 1979		√
				*Parmaturus xaniurus* * (Gilbert, 1892)	√	
		Scyliorhinidae	*Cephaloscyllium*	*Cephaloscyllium ventriosum* * (Garman, 1880)	√	
			*Scyliorhinus*	*Scyliorhinus hesperius* Springer, 1966		√
				*Scyliorhinus meadi* Springer, 1966		√
				*Scyliorhinus retifer* (Garman, 1881)		√
		Sphyrnidae	*Sphyrna*	*Sphyrna corona* Springer, 1940	√	
				*Sphyrna lewini* * (Griffith & Smith, 1834)	√	√
				*Sphyrna media* Springer, 1940	√	
				*Sphyrna mokarran* (Rüppell, 1837)	√	√
				*Sphyrna tiburo* (Linnaeus, 1758)	√	√
				*Sphyrna zygaena* * (Linnaeus, 1758)	√	√
		Triakidae	*Galeorhinus*	*Galeorhinus galeus* (Linnaeus, 1758)	√	
			*Mustelus*	*Mustelus albipinnis* * Castro-Aguirre, Antuna-Mendiola, González-Acosta & De La Cruz-Agüero, 2005	√	
**Selachii**	**Carcharhiniformes**	Triakidae	*Mustelus*	*Mustelus californicus* * Gill, 1864	√	
				*Mustelus canis* (Mitchill, 1815)		√
				*Mustelus dorsalis* Gill, 1864	√	
				*Mustelus henlei* * (Gill, 1863)	√	
				*Mustelus higmani* Springer & Lowe, 1963		√
				*Mustelus lunulatus* * Jordan & Gilbert, 1882	√	
				*Mustelus norrisi* Springer, 1939		√
				*Mustelus sinusmexicanus* Heemstra, 1997		√
			*Triakis*	*Triakis semifasciata* * Girard, 1855	√	
	**Hexanchiformes**	Chlamydoselachidae	*Chlamydoselachus*	*Chlamydoselachus anguineus* Garman, 1884	√	√
		Hexanchidae	*Heptranchias*	*Heptranchias perlo* (Bonnaterre, 1788)		√
			*Hexanchus*	*Hexanchus griseus* * (Bonnaterre, 1788)	√	
				*Hexanchus nakamurai* Teng, 1962		√
				*Hexanchus vitulus* Springer & Waller, 1969		√
			*Notorynchus*	*Notorynchus cepedianus* (Péron, 1807)	√	
	**Squaliformes**	Centrophoridae	*Centrophorus*	*Centrophorus granulosus* (Bloch & Schneider, 1801)		√
				*Centrophorus uyato* (Rafinesque, 1810)		√
		Dalatiidae	*Dalatias*	*Dalatias licha* (Bonnaterre, 1788)		√
			*Euprotomicrus*	*Euprotomicrus bispinatus* (Quoy & Gaimard, 1824)		√
			*Isistius*	*Isistius brasiliensis* (Quoy & Gaimard, 1824)	√	√
				*Isistius plutodus* Garrick & Springer, 1964		√
			*Squaliolus*	*Squaliolus laticaudus* Smith & Radcliffe, 1912		√
		Etmopteridae	*Centroscyllium*	*Centroscyllium nigrum* * Garman, 1899		√
			*Etmopterus*	*Etmopterus bullisi* Bigelow & Schroeder, 1957		√
				*Etmopterus hillianus* (Poey, 1861)		√
				*Etmopterus schultzi* Bigelow, Schroeder & Springer, 1953		√
				*Etmopterus virens* Bigelow, Schroeder & Springer, 1953	√	
		Oxynotidae	*Oxynotus*	*Oxynotus caribbaeus* Cervigón, 1961		√
		Somniosidae	*Centroscymnus*	*Centroscymnus coelolepis* Barbosa du Bocage & de Brito Capello, 1864		√
**Selachii**	**Squaliformes**	Somniosidae	*Centroscymnus*	*Centroscymnus owstonii* Garman, 1906		√
			*Somniosus*	*Somniosus microcephalus* (Bloch & Schneider, 1801)		√
				*Somniosus pacificus* Bigelow & Schroeder, 1944	√	
			*Zameus*	*Zameus squamulosu*s (Günther, 1877)		√
		Squalidae	*Cirrhigaleus*	*Cirrhigaleus asper* (Merrett, 1973)		√
			*Squalus*	*Squalus acanthias* * Linnaeus, 1758	√	√
				*Squalus cubensis* Howell Rivero, 1936		√
				*Squalus mitsukurii* Jordan & Snyder, 1903		√
				*Squalus suckleyi* (Girard, 1855)	√	
	**Echinorhiniformes**	Echinorhinidae	*Echinorhinus*	*Echinorhinus brucus* (Bonnaterre, 1788)		√
				*Echinorhinus cookei* Pietschmann, 1928	√	
	**Squatiniformes**	Squatinidae	*Squatina*	*Squatina californica* * Ayres, 1859	√	
				*Squatina dumeril* Lesueur, 1818		√
				*Squatina heteroptera* ? Castro-Aguirre, Espinosa Pérez & Huidobro Campos, 2007		√
				*Squatina mexicana* ? Castro-Aguirre, Espinosa Pérez & Huidobro Campos, 2007		√
**Batomorphi**	**Rajiformes**	Anacanthobatidae	*Springeria* †	*Springeria folirostris* (Bigelow & Schroeder, 1951)		√
				*Springeria longirostris* Bigelow & Schroeder, 1962		√
		Arhynchobatidae	*Bathyraja*	*Bathyraja abyssicola* (Gilbert, 1896)	√	
				*Bathyraja interrupta* (Gill & Townsend, 1897)	√	
				*Bathyraja spinosissima* (Beebe & Tee-Van, 1941)	√	
				*Bathyraja trachura* (Gilbert, 1892)	√	
			*Pseudoraja*	*Pseudoraja fischeri* Bigelow & Schroeder, 1954		√
		Gurgesiellidae †	*Cruriraja*	*Cruriraja poeyi* Bigelow & Schroeder, 1948		√
				*Cruriraja rugosa* Bigelow & Schroeder, 1958		√
			*Fenestraja*	*Fenestraja ishiyamai* (Bigelow & Schroeder, 1962)		√
				*Fenestraja plutonia* (Garman, 1881)		√
				*Fenestraja sinusmexicanus* (Bigelow & Schroeder, 1950)		√
**Batomorphi**	**Rajiformes**	Gurgesiellidae †	*Gurgesiella*	*Gurgesiella atlantica* (Bigelow & Schroeder, 1962)		√
		Rajidae	*Amblyraja*	*Amblyraja badia* (Garman, 1899)	√	
				*Amblyraja hyperborea* (Collett, 1879)	√	
			*Beringraja*	*Beringraja binoculata* (Girard, 1855)	√	
				*Beringraja cortezensis* † (McEachran & Miyake, 1988)	√	
				*Beringraja inornata* †* (Jordan & Gilbert, 1881)	√	
				*Beringraja rhina* † (Jordan & Gilbert, 1880)	√	
				*Beringraja stellulata* † (Jordan & Gilbert, 1880)	√	
			*Breviraja*	*Breviraja colesi* Bigelow & Schroeder 1948		√
				*Breviraja spinosa* Bigelow & Schroeder, 1950		√
			*Dactylobatus*	*Dactylobatus armatus* Bean & Weed, 1909		√
				*Dactylobatus clarkii* (Bigelow & Schroeder, 1958)		√
			*Dipturus*	*Dipturus bullisi* (Bigelow & Schroeder, 1962)		√
				*Dipturus garricki* (Bigelow & Schroeder, 1958)		√
				*Dipturus olseni* (Bigelow & Schroeder, 1951)		√
				*Dipturus oregoni* (Bigelow & Schroeder, 1958)		√
				*Dipturus teevani* (Bigelow & Schroeder, 1951)		√
			*Leucoraja*	*Leucoraja garmani* (Whitley, 1939)		√
				*Leucoraja lentiginosa* (Bigelow & Schroeder, 1951)	√	
				*Leucoraja yucatanensis* (Bigelow & Schroeder, 1950)		√
			*Rajella*	*Rajella fuliginea* (Bigelow & Schroeder, 1954)		√
				*Rajella purpuriventralis* † (Bigelow & Schroeder, 1962)		√
			*Rostroraja* †	*Rostroraja ackleyi* (Garman, 1881)		√
				*Rostroraja eglanteria* (Bosc, 1800)		√
				*Rostroraja equatorialis* * (Jordan & Bollman, 1890)	√	
				*Rostroraja texana* (Chandler, 1921)		√
				*Rostroraja velezi* (Chirichigno, 1973)	√	
	**Torpediniformes**	Narcinidae	*Diplobatis*	*Diplobatis ommata* * (Jordan & Gilbert, 1890)	√	
			*Narcine*	*Narcine bancroftii* (Griffith & Smith, 1834)		√
				*Narcine brasiliensis* (Olfers, 1831)		√
**Batomorphi**	**Torpediniformes**	Narcinidae	*Narcine*	*Narcine entemedor* * Jordan & Starks, 1895	√	
				*Narcine vermiculatus* Breder, 1928	√	
		Torpedinidae	*Tetronarce*	*Tetronarce californica* (Ayres, 1855)	√	
				*Tetronarce nobiliana* (Bonaparte, 1835)		√
			*Torpedo*	*Torpedo andersoni* Bullis, 1962		√
		Platyrhinidae	*Platyrhinoidis*	*Platyrhinoidis triseriata* * (Jordan & Gilbert, 1880)	√	
	**Rhinopristiformes** †	Pristidae	*Pristis*	*Pristis pectinata* Latham, 1794		√
				*Pristis pristis* (Linnaeus, 1758)	√	√
		Rhinobatidae	*Pseudobatos* †	*Pseudobatos glaucostigmus* * (Jordan & Gilbert, 1883)	√	
				*Pseudobatos lentiginosus* (Garman, 1880)		√
				*Pseudobatos leucorhynchus* * (Günther, 1866)	√	
				*Pseudobatos percellens* (Walbaum, 1792)		√
				*Pseudobatos planiceps* (Garman, 1880)	√	
				*Pseudobatos prahli* (Acero & Franke, 1995)	√	
				*Pseudobatos productus* * (Ayres, 1854)	√	
				*Pseudobatos spinosus* ? (Günther, 1870)	√	
		Trygonorrhinidae †	*Zapteryx*	*Zapteryx exasperata* * (Jordan & Gilbert, 1880)	√	
				*Zapteryx xyster* Jordan & Evermann, 1896	√	
	**Myliobatiformes**	Aetobatidae †	*Aetobatus*	*Aetobatus narinari* (Euphrasen, 1790)		√
				*Aetobatus laticeps* * † (Gill, 1865)	√	
		Dasyatidae	*Hypanus* †	*Hypanus americanus* (Hildebrand & Schroeder, 1928)		√
				*Hypanus dipterurus* * (Jordan & Gilbert, 1880)	√	
				*Hypanus guttatus* (Bloch & Schneider, 1801)		√
				*Hypanus longus* (Garman, 1880)	√	
				*Hypanus sabinus* (Lesueur, 1824)		√
				*Hypanus say* (Lesueur, 1817)		√
			*Pteroplatytrygon*	*Pteroplatytrygon violacea* (Bonaparte, 1832)	√	√
		Potamotrygonidae	*Styracura* †	*Styracura pacifica* (Beebe & Tee-Van, 1941)	√	
				*Styracura schmardae* (Werner, 1904)	√	√
**Batomorphi**	**Myliobatiformes**	Gymnuridae	*Gymnura*	*Gymnura crebripunctata* * (Peters, 1869)	√	
				*Gymnura marmorata* * (Cooper, 1864)	√	
				*Gymnura lessae* Yokota & De Carvalho, 2017		√
		Mobulidae	*Mobula*	*Mobula alfredi* † (Krefft, 1868)		√
				*Mobula birostris* † (Walbaum, 1792)	√	√
				*Mobula hypostoma* (Bancroft, 1831)		√
				*Mobula mobular* (Bonnaterre, 1788)	√	
				*Mobula munkiana* Notarbartolo Di Sciara, 1987	√	
				*Mobula tarapacana* (Philippi, 1892)	√	
				*Mobula thurstoni* (Lloyd, 1908)	√	
		Myliobatidae	*Myliobatis*	*Myliobatis californica* * Gill, 1865	√	
				*Myliobatis longirostris* * Applegate & Fitch, 1964	√	
			*Aetomylaeus*	*Aetomylaeus asperrimus* (Gilbert, 1898)	√	
		Rhinopteridae	*Rhinoptera*	*Rhinoptera bonasus* (Mitchill, 1815)		√
				*Rhinoptera brasiliensis* Müller, 1836		√
				*Rhinoptera steindachneri* * Evermann & Jenkins, 1891	√	
		Urotrygonidae	*Urobatis*	*Urobatis concentricus* * Osburn & Nichols, 1916	√	
				*Urobatis halleri* * (Cooper, 1863)	√	
				*Urobatis jamaicensis* (Cuvier, 1816)		√
				*Urobatis maculatus* * Garman, 1913	√	
			*Urotrygon*	*Urotrygon aspidura* * (Jordan & Gilbert, 1882)	√	
				Urotrygon chilensis* (Günther, 1872)	√	
				*Urotrygon cimar* López & Bussing, 1998	√	
				*Urotrygon munda* Gill, 1863	√	
				*Urotrygon nana* Miyake & McEachran, 1988	√	
				*Urotrygon rogersi* * (Jordan & Starks, 1895)	√	
				*Urotrygon simulatrix* Miyake & McEachran, 1988	√	

Field-specific books were consulted (e.g., Compagno 1999, Castro 2011a, Ebert et al. 2013, Last et al. 2016a) for comparative purposes and several recent scientific publications were reviewed in a deliberate manner (e.g., Ruiz-Campos et al. 2010, Hoyos-Padilla et al. 2013, Del Moral-Flores et al. 2015a, Del Moral-Flores et al. 2016, Villalobos et al. 2016, Weigmann 2016, 2017). In this way, a clear, cumulative database was compiled with all existing records for species within the Mexican EEZ. This database was cross-referenced with information available online from biological collections and museums worldwide. Global databases consulted included the Ocean Biogeographic Information System (OBIS 2017), the Global Biodiversity Information Facility (GBIF 2017), the Encyclopedia of Life (EOL 2017), iSpecies (2017), the FishBase Project (Froese and Pauly 2017) and the National Biodiversity Information System (SNIB–CONABIO, https://goo.gl/9aNLSv).

Chondrichthyes taxa were verified using published references from online resources (i.e., the Internet) such as the Catalog of Fishes of the California Academy of Sciences (https://goo.gl/S792vp), World Register of Marine Species (http://www.marinespecies.org/), Integrated Taxonomic Information System (https://www.itis.gov/), and Chondrichthyan Tree of Life (https://sharksrays.org/). Online database searches were carried out between June 22 and November 15 of 2017. The systematic arrangement (Table 1) is organized in a manner based on the proposal in Weigmann (2016) with some modifications for the group of skates and rays species (Last et al. 2016a). Finally, the spellings in the citations of the common names of the species (sensu Froese and Pauly 2017) follows Nelson et al. (2002).

## Results

The detailed literature review involving the species of chimaeras, sharks, skates, and rays that currently exist worldwide includes 1,212 species (Weigmann 2017). Taxa delineations are in accordance with the phylogenetic system published by Weigmann (2016) for the Chondrichthyes and include two subclasses (Holocephali and Euselachii), one infraclass (Elasmobranchii) with two divisions (Selachii and Batomorphi), 14 orders, 64 families, and 215 genera. The phylogeny includes one order, three families, six genera, and 49 species of chimaeras; nine orders, 34 families, 105 genera, and 517 species of sharks; and four orders, 27 families, 104 genera, and 646 species of skates and rays. For a comparison of the number of species of Chondrichthyes recognized worldwide between 2005 and 2015, see Table 1 of Weigmann (2016).

It should be noted that the specific taxonomic richness of the Chondrichthyes of Mexico may vary slightly within the recent literature (e.g., Weigmann 2016, Last et al. 2016a, Del Moral-Flores et al. 2015a) (approximately ten or fewer species). However, at the family level for the batomorphs, there are important taxonomic implications with a difference of up to nine families. Thus, the proposed classification for the skates and rays by Last et al. (2016a) was included in this systematic listing for the Mexican EEZ, regarding the newest taxa in the Dasyatidae family (Last et al. 2016b), the resurrection of the Aetobatidae family (White and Naylor 2016), the new order Rhinopristiformes (Last et al. 2016c), and nomenclatural changes made in the order Rajiformes (Last et al. 2016d). For a detailed description of the taxonomic changes in Chondrichthyes in general, see Weigmann (2017).

The dynamism (or uncertainty) of the classification of Chondrichthyes can be exemplified by the Thornback guitarfish, *Platyrhinoidis triseriata* (Jordan & Gilbert, 1880), which is currently recognized in two families, Rhinobatidae (sensu Froese and Pauly 2017) and Platyrhinidae (sensu Last et al. 2016a), and in four different orders such as, Rajiformes (Froese and Pauly 2017), Rhinobatiformes (Weigmann 2016), Torpediniformes (Last et al. 2016a) and Myliobatiformes (Nelson et al. 2016). Herein, for the species mentioned, we follow the criteria for family allocation of Naylor et al. (2012) based on DNA sequences, which concurs with the criteria that the American zoologist Samuel W. Garman suggested more than a century ago (Garman 1913: 290). At the order level, we adopted the proposal depicted in the phylogenetic tree by Yang et al. (unpublished) cited in Last et al. (2016a) followed too, in the Chondrichthyan Tree of Life Project (https://sharksrays.org/).

Finally, according to the worldwide species richness of Chondrichthyes, in Mexico, there are no records of one family of Holocephali, the Plownose chimaeras, Callorhinchidae (distributed in the south of the American continent); neither for 11 families of sharks: Parascylliidae, Brachaeluridae, Orectolobidae, Hemiscylliidae, Stegostomatidae, Leptochariidae, Hemigaleidae, Pseudotriakidae, Pristiophoridae, Proscylliidae, and Mitsukurinidae (the last three families having species that inhabit the American continent and are likely to be found in the future within the Mexican EEZ, such as the Goblin shark *Mitsukurina owstoni* Jordan, 1898); and finally not for eight families of skates and rays: Glaucostegidae, Hexatrygonidae, Hypnidae, Rhinidae, Narkidae, Plesiobatidae, Urolophidae and Zanobatidae (none of which are distributed in the American continent).

### The Mexican Exclusive Economic Zone (EEZ)

A total of 217 species of chimaeras, sharks, skates, and rays was recorded and classified into two subclasses, one infraclass, two divisions, 13 orders, 44 families, and 84 genera (Table 1). This species richness corresponds to 17.9% of the total of current species of Chondrichthyes worldwide, according to Weigmann (2017). The Euselachii subclass was the most representative in terms of species richness at 96.3%, while the Holocephali subclass was the least represented at only 3.7%. The chimaeras were represented in the Mexican EEZ exclusively by a single order (Chimaeriformes) with two families. Of these, Rhinochimaeridae was the best represented with two genera, while *Hydrolagus* (Chimaeridae) was the genus that had the largest number of species (five species). In terms of sharks, the Carcharhiniformes and Squaliformes orders were represented by six families each. Within Carcharhiniformes, the Carcharhinidae family contained seven genera, and *Carcharhinus* had 18 species resulting with the highest species diversity. Finally, for rays and skates, the order Myliobatiformes was represented by eight families, where the genera *Pseudobatos* (eight species), *Mobula* (seven species) and *Urotrygon* (seven species) were the most diverse at the species level. On the other hand, the family Rajidae (order Rajiformes) contained eight different genera with 27 species (Table 1).

### The Pacific Ocean and the Gulf of California (west coast)

For this area of the Mexican EEZ, Chondrichthyes was represented by 92 species found only in this area and 31 species distributed on both coasts (i.e., with an amphi-American distribution), for 123 species. These species belong to two subclasses (Table 1), with sharks (Selachii division) representing 51.2% of the species (63 species), followed by rays (Batomorphi division) representing 44.7% of the species (55 species) and finally chimaeras (subclass Holocephali), with 4.1% of the species (five species). Considering both coasts of Mexico, the species richness for this zone represents 49.59% of the total of Chondrichthyes currently recorded for the EEZ (of a total of 248 species including the amphi-American species).

The order Chimaeriformes was represented by two families (Chimaeridae and Rhinochimaeridae) with two genera and five species, representing 62.5% of the chimaera species reported for Mexico (Table 1). The two species of chimaeras from the genus *Harriotta* (Rhinochimaeridae) were distributed exclusively on this basin of the Mexican EEZ.

For the group of sharks, eight different orders were recorded, including 22 families, 36 genera, and 63 species. The Carcharhiniformes were the best represented with six families. Specifically, Carcharhinidae was the most taxonomically diverse with seven genera and 17 species. Of these species, eleven belong to the genus *Carcharhinus*. The Triakidae family includes three genera and seven species, which five species belonged to the genus *Mustelus* (Table 1). The horn sharks, Heterodontidae family, were distributed exclusively within the Pacific Mexican EEZ. On the other hand, the batomorphs were represented by four orders, 16 families, 23 genera, and 55 species (Table 1). The genus *Urotrygon* (Myliobatiformes: Urotrygonidae) contained the highest diversity of batomorph, with seven species (Table 1). The Banded guitarfish *Zapteryx exasperata* (Jordan & Gilbert, 1880) and the Witch guitarfish *Z. xyster* (Jordan & Evermann, 1896) are now members of the new family Trygonorrhinidae (sensu Last et al. 2016c), and they are exclusively distributed in the western slope of the Mexican EEZ.

In this area, the following species were identified as endemic: the Whitemargin smoothhound *Mustelus albipinnis* Castro-Aguirre, Antuna-Mendiola, González-Acosta & De La Cruz-Agüero, 2005 (Carcharhiniformes: Triakidae), the Spotted round ray *Urobatis maculatus* Garman, 1913 (Myliobatiformes: Urotrygonidae), the Cortez skate *Beringraja cortezensis* (McEachran & Miyake, 1988) (Rajiformes: Rajidae) and the Spiny guitarfish *Pseudobatos spinosus* (Günther, 1870) (Rhinopristiformes: Rhinobatidae). For the latter species, see the Discussion.

### The Gulf of Mexico and the Caribbean Sea (east coast)

The Exclusive Economic Zone of the eastern slope of Mexico was represented by 94 chondrichthyans species that occur only in this area and 31 amphi-American species (125 in total), belonging to two subclasses (Table 1). Of these, 75 species belong to sharks (60.0%), 47 (37.6%) to skates and rays, and three (2.4%) to chimaeras. Considering both coasts of Mexico, the species richness for this zone represents 50.41% of the total chondrichthyans currently recorded (of 248 species including the amphi-Americans species).

The Holocephali were grouped into one order (Chimaeriformes), two families, two genera, and three species that represent approximately 40% of the species recorded to date for the Mexican EEZ (Table 1). Within the sharks, the Squaliformes and Lamniformes orders contained the most number of families with six families. However, the Carcharhinidae family (order Carcharhiniformes) had the highest number of genera with five genera, and the *Carcharhinus* genus had the highest species richness with 14 species (Table 1). The sharks belonging to the family Centrophoridae were distributed exclusively in this slope of the EEZ of Mexico. For the batomorphs, the order Myliobatiformes contained the largest number of families with seven, where Rajidae with six genera was the most diverse, as its genus *Dipturus* is the taxon with the highest species richness with five species (Table 1).

For the Gulf of Mexico and the Caribbean Sea there were three endemic species of sharks: Campeche catshark *Parmaturus campechiensis* Springer 1979 (Carcharhiniformes: Scyliorhinidae), the Disparate angel shark *Squatina heteroptera* Castro-Aguirre, Espinosa Pérez & Huidobro Campos, 2007 the Mexican angel shark *S. mexicana* Castro-Aguirre, Espinosa Pérez & Huidobro Campos, 2007 (Squatiniformes: Squatinidae) and one species of skate, the Yucatan skate *Leucoraja yucatanensis* (Bigelow & Schroeder, 1950). However, see in Discussion about these Angel shark species.

### Amphi-American Chondrichthyes in the EEZ of Mexico

Thirty-one species of chondrichthyans were recorded on both oceanic basins of the EEZ of Mexico. Sharks, 27 species, constituted approximately 87% of the total of species followed by four species of rays. Amphi-American chimaeras were not recorded.

Among the sharks, the genus *Carcharhinus* (Carcharhiniformes: Carcharhinidae) is the richest with seven species, the Bull shark *C. leucas* (Müller & Henle, 1839), the Oceanic whitetip shark *C. longimanus* (Poey, 1861), the Blacktip shark *C. limbatus* (Müller & Henle, 1839), the Silky shark *C. falciformis* (Müller & Henle, 1839), the Dusky shark *C. obscurus* (Lesueur, 1818), the Smalltail shark *C. porosus* (Ranzani, 1839) and the Bignose shark *C. altimus* (Springer, 1950). The hammerhead sharks of the Sphyrnidae family with distributions on both coasts are the Scalloped hammerhead *Sphyrna lewini* (Griffith & Smith, 1834), the Great hammerhead *S. mokarran* (Rüppell, 1837), the Bonnethead *S. tiburo* (Linnaeus, 1758) and the Smooth hammerhead *S. zygaena* (Linnaeus, 1758).

In the case of the batomorphs, only four species are distributed on both coasts of the country, the Pelagic stingray *Pteroplatytrygon violacea* (Bonaparte, 1832), the Chupare stingray *Styracura schmardae* (Werner, 1904), the Giant manta *Mobula birostris* (Walbaum, 1792) and the Common sawfish *Pristis pristis* (Linnaeus, 1758). The amphi – American distribution of the species *Ginglymostoma cirratum* Bonnaterre, 1788 (Nurse shark) and *Aetobatus narinari* (Euphrasen, 1790) (the Spotted eagle ray) is not considered in the present study because of the reasons stated in the Discussion.

## Discussion

The species richness of Mexican chimaeras, sharks, skates, and rays, when compared to other Latin American countries, is above the 165 species reported for Brazil (Rosa and Gadig 2014); the 117 from Colombia (62 Pacific coast species and 75 for the Caribbean Sea; Navia et al. 2016); the 99 for Costa Rica (12 Caribbean Sea, 75 Pacific, and 12 amphi-American; Bussing and López 1999,^2010^, Espinoza et al. 2018); the 98 for Venezuela (60 sharks, 37 rays, and one chimera; Cervigón and Alcalá 1999, Tavares and López 2009), and the 38 species for Ecuador (Jiménez-Prado and Beárez 2004, Estupiñan-Montaño et al. 2016). This diversity of Chondrichthyes inhabiting the EEZ of Mexico (approximately 3,000,150 km^2^) makes it a megadiverse country for this group of species.

The total numbers of chondrichthyans fishes herein reported to the species, genus, family, and order levels in this study are 217, 84, 44, and 13, respectively. These numbers are similar to those reported for Mexico by Del Moral-Flores et al. (2015a) and Del Moral-Flores et al. (2016). However, some of the discrepancies can be attributed to recent taxonomic readjustments, especially for the Division Batomorphi.

Sharks, in general, are the group with the highest diversity of species in the EEZ of Mexico with 111 species (51%). These results are consistent with a previous study by Del Moral-Flores et al. (2016), who reported the same number of species; however, the systematic inventories are not equivalent because the present study does not include records of species reported by those authors as *aff. sp.* or species herein recognized as synonyms (e.g., *Sphyrna vespertina* Springer, 1940; *Negaprion fronto* (Jordan & Gilbert, 1882); *Centrophorus niaukang* Teng, 1959) or subspecies (e.g. *Leucoraja garmani caribbaea* (McEachran, 1977)).

The group of the skates and rays contained 98 species and constituted 45% of the total diversity recorded for the EEZ of Mexico. This figure is very similar to that reported by Del Moral-Flores et al. (2016), who listed 95 species. The species additions to the Mexican chondrichthyans for this group are the Longsnout butterfly ray *Gymnura crebripunctata* (Peters, 1869), the Lessa´s butterfly ray *Gymnura lessae* Yokota & De Carvalho, 2017 (see below), the Brazilian cownose ray *Rhinoptera brasiliensis* Müller, 1836, the Fake round ray *Urotrygon simulatrix* Miyake & McEachran, 1988, and the Pacific guitarfish *Pseudobatos planiceps* (Garman, 1880) (the latter species is placed in a new genus, sensu Last et al. 2016c). The eight species of chimaeras did not present any differences among the bibliographic sources and the databases consulted.

Although the Batomorphi constitutes approximately 53% (633) of the total living species of the Chondrichthyes class worldwide, with an addition of at least fifty detected species and yet to be described (Last et al. 2016a), it is not surprising that sharks are the most representative group in the present study. This phenomenon is considered to be related to a historical preference for studying sharks, due to their charismatic characteristics (sensu Isasi-Catalá 2011), and a greater interest in fishing for them, in comparison with the rays and skates. However, currently, the number of scientists referencing batomorphs is increasing, allowing us to consider a scenario where skates and rays species might be studied with a greater emphasis at national and international levels (see Last et al. 2016e).

The new families identified and restored by Last et al. (2016c) and White and Naylor (2016) (Trygonorrhinidae and Aetobatidae, respectively) are represented within the EEZ of Mexico, with the records of the species of guitarfish *Zapteryx exasperata* and *Z. xyster* (Rhinopristiformes: Trygonorrhinidae) for the coasts of the Pacific Ocean and the Eagle rays *Aetobatus narinari* and *A. laticeps* (Gill, 1865) (Myliobatiformes: Aetobatidae) for the Atlantic and Pacific Oceans, respectively. Recently, the existence of genetic and morphometric variability in a latitudinal gradient for the guitar species of the *Zapteryx* genus has been identified and should be studied in more detail (Castillo-Páez et al. 2017).

According to the information in OBIS (2017) and GBIF (2017), the Reef stingray *Urobatis concentricus* Osburn & Nichols, 1916 (Myliobatiformes: Urotrygonidae) is exclusive to the western Mexican seas of the Mexican EEZ except for three records from Costa Rica. We considered that these latter records are misidentification or a data capture error in the computer platforms previously mentioned. Thus, in the study conducted by Palacios-Salgado et al. (2012), they do not consider this species (or any other batomorph species) as endemic to the Cortez biogeographic province (eastern Pacific Ocean Region, sensu Briggs (1974)). Nevertheless, other authors consider this species as endemic to the western coast of Mexico (Ehemann et al. 2017).

In the case of the Bat ray *Myliobatis californica* (Gill, 1865) (Myliobatiformes: Myliobatidae), a situation similar to that previously reported for *U. concentricus* occurred, because Palacios-Salgado et al. (2012) also make no reference to this species as endemic to the Cortez biogeographic province as there are records outside the study area of the authors. The source of the data consulted (i.e., OBIS 2017) reviewed all the records for this species (n = 73) within the continental shelf of Mexico, which may be considered as an endemic species. However, another source of data consulted (i.e., GBIF 2017) presented more than 500 records, with a few dozen registered for the area of the United States of America, and three records for Indonesia, the Maldives and Panama, which could be attributed, again, to misidentification or a capture error within the GBIF platform, which would corroborate this hypothesis with the one record of this species in the Gulf of Mexico.

The Spiny guitarfish *Pseudobatos spinosus* (Günther, 1870) (Rhinopristiformes: Rhinobatidae) had a single record in the databases consulted (i.e., OBIS 2017), which referred to the collection location of the holotype. Excluding the original description and some of the checklist and computer databases references (e.g., Del Moral-Flores et al. 2015a, Del Moral-Flores et al. 2016, Froese and Pauly 2017), there is no other information available for this species. According to F. Del Moral-Flores (UNAM–Campus Iztacala, pers. comm.), the record could be related to an anomalous specimen (i.e., the holotype) of *Pseudobatos* spp., described as *Rhinobatos spinosus* by Günther (1870: 518). Another possibility that has been cited for this record is that it may be a juvenile specimen of another Rhinobatidae species (Compagno 1999: 471–498). Taking into consideration the conditions of the holotype (a dissected and unrecognizable specimen of 33 cm in length, deposited in the British Museum, BMNH: 1870.6.20.2) and the total absence of records since its diagnosis, the exclusion of this species from the EEZ of Mexico could be considered. Thus, the taxonomic lists and identification guides would avoid, to some extent, the overestimation of the chondrichthyans species from the EEZ of Mexico.

For the Disparate angel Shark (*Squatina heteroptera*) and the Mexican angel shark (*S. mexicana*) (both referred as endemic species from the east coast of Mexico), recently Weigmann (2016) and Vaz and De Carvalho (2018), have treated these species as junior synonyms of *Squatina dumeril* Lesueur, 1818. Despite the fact that those authors came to the same preliminary conclusion, in this manuscript each *Squatina* species mentioned, are retained as valid species. Nevertheless, it is highly recommended to do further investigation to demonstrate its taxonomic validity. Such as the Spiny guitarfish, these two species are marked in the checklist with a question mark (i.e. species whose validity requires further investigation).

At present, there are recent publications that support the separation of a species considered to have an amphi-American distribution, which is the case for the Nurse shark *Ginglymostoma cirratum* Bonnaterre, 1788 and the UNAM´s nurse shark *Ginglymostoma unami* Del Moral-Flores, Ramírez-Antonio, Angulo & Pérez-Ponce de León, 2015. The latter species was described from specimens collected in the tropical eastern Pacific Ocean initially identified as *G. cirratum* and recorded as this new species (*G. unami*) and as endemic to this region, excluding the presence of *G. cirratum* for the Pacific Ocean (Del Moral-Flores et al. 2015b).

Another similar case is for the species the Spotted eagle ray *Aetobatus narinari* (Euphrasen, 1790), considered as an amphi-American species (in fact cosmopolitan species). Currently, based on DNA sequences (White and Naylor 2016, Last et al. 2016a), the Pacific eagle ray *Aetobatus laticeps* is recognized as distributed exclusively on the eastern Pacific coast, while its congener *A. narinari* is an inhabitant of the western Atlantic coast including the Gulf of Mexico and the Caribbean Sea. However, Last et al. (2016e) consider “that it is necessary to do more work to distinguish morphologically the two forms”. Currently, the species is cited as an ambiguous synonym for *A. narinari* in Froese and Pauly (2017).

According to the recent morphometric and molecular results obtained by De Carvalho et al. (2016), the subfamily Styracurinae was described and relocated within the family Potamotrygonidae, which are freshwater batomorphs known only in South America until these recent results. The species *Styracura scharmardae* (Werner, 1904) and the Pacific chupare ray *S. pacifica* (Beebe & Tee-Van, 1941) were removed from *Himantura* in Dasyatidae (sensu Last et al. 2016c, De Carvalho et al. 2016) and are thus the only representatives of the potamotrygonids within the EEZ of Mexico; the first species is cited an inhabitant of both coasts, and the second species is restricted to the western basin.

The recent taxonomic relocation of the two species of the genus *Manta*, the Reef manta ray *M. alfredi* (Krefft, 1868) and the Giant oceanic manta ray *M. birostris* (Walbaum, 1792) within the genus *Mobula* (see Last et al. 2016a), and the consideration of the Spinetail mobula *Mobula japanica* (Müller & Henle, 1841) as synonymous with the Devil fish *M. mobular* (Bonnaterre, 1788), are considered by some specialists as a taxonomic decision subject to discussion (Guy Stevens, The Manta Team: https://goo.gl/KYbtZO; com. pers.).

Finally, a recent taxonomic and morphological revision of butterfly rays (Gymnuridae) has limited the distribution of the Smooth butterfly ray *Gymnura micrura* (Bloch & Schneider, 1801) to the southwestern Atlantic and the new species the Lessa´s butterfly ray *Gymnura lessae* Yokota & De Carvalho, 2017 occurring in the Gulf of Mexico, north, and central western Atlantic, substituting *G. micrura* records in that area (see Yokota and De Carvalho 2017).

With the increasing use of various techniques and the analysis tools currently available (e.g., molecular sequences, mitogenome analysis, geometric morphometrics), the future of the biological classification of Chondrichthyes may have higher stability, predictability, and robustness (sensu Crisci and López-Armengol 1983). Due to its geographical location, the extension of its patrimonial sea, and the increase in studies on its chondrichthyans, Mexico will undoubtedly continue to contribute to the knowledge for this group of cartilaginous fishes. At present, for the country, there are at least four species of sharks and two batomorphs that need to be formally described, which have been previously mentioned by various authors (e.g., Castro-Aguirre et al. 1996, Castro 2011a, 2011b, Del Moral-Flores et al. 2015a).

As a corollary to the above, recently published works or studies in progress can be cited. Thus, Fields et al. (2016) proposed the existence of populations with possible cryptic speciation among hammerhead sharks (Sphyrnidae) from the Gulf of Mexico and the Caribbean Sea. Another study conducted by Hinojosa-Alvarez et al. (2016) indicates the possibility of a third species of manta ray within the genus *Mobula* (sensu lato *Manta*, see Last et al. 2016a) for the Yucatan Peninsula. Finally, the mitochondrial divergence between the populations of the Cownose ray *Rhinoptera steindachneri* Evermann & Jenkins, 1891, of the Gulf of California (see also Sandoval-Castillo and Rocha-Olivares 2011) is currently being investigated (Christian Jones, NOAA–SFSC Mississippi, pers. comm.), as is the case of *Urotrygon* spp. in the southern Gulf of California by the present authors, what could result in the description of new species in the EEZ of Mexico.
